# Supplementary material for: Chromothripsis during telomere crisis is independent of NHEJ, and consistent with a replicative origin
Source: Genome Res. 2019 May;29(5):737–49. doi: 10.1101/gr.240705.118 (PMC6499312; doi:10.1101/gr.240705.118)
Supplement: Supplemental Material [file supp_gr.240705.118_Supplemental_file_1.zip › contigs/annotated_contigs/DB46/contig.2.DB46_length_714_mean_cov_6.8431372549.docx]

**DB46_length_714_mean_cov_6.8431372549**

```
TTCTTGAAGAATATTAACAAATGGACAAAACTCTGGCCTGACTGATTTAAGAGAAAAGGGAAAAATACCAAGTACAAATCTCAAAACCG
 >chr2:15685505-15685941 + E=1e-250 p=7e-03
ATACAGGGGACGTCAGTACAGATCCTACAGACAGAAGGGTAATAGGGAATATTATAAACATTAAGCCAATAATTTCTACAATTTAGGGA

AAAATTCCACAAAAACTGAAATAAAAGGAAACCAAAATTTTAAAAAGCCTTATTAATTACTAGTATTTTAACTAAACTCAAGATTTAAA

ATTGGTTAATTAAAAGACTGCTTACAAGAAAACTCCAAGTTTCACTGGTGAATTCCATCAAACATTTAAGGAAGAAATAATTCAATTCT

ACACAAATTGTTTCAGAAAATAAAAAAGGAGGGAACACTTGCCAACCCTTTTCAAACCAGCACTACTCCAATACC|AAACC|CCGACTC
                                                                            >chr2:15686
TACTAAAAACACAAAAATTAGCTGGGCATGGTGGCAGGTGCCTATAATCCCAGCTACTTGGAGTCTGAGGCAGGAGAATCACTTGAACT
674-15686957 + E=2e-158
CGGGAGGTGGAGGTTGCAGTGAGCCTAGATGGCGCCACTGCACTCCAGCCTCAGTGACAGAGCCAGACTCCGTCTGAAAACAAAACAAA

AAAAACAAGTCTGGGTGTTTCAAATAGTCTATCCTTCCCAACCTTTTTCATGTCAACACACAGAGAAAATCATATTTGTATGGCATCTA

ACAG
```
